# Case report: 3D intracranial vessel wall MRI in Susac syndrome: potential relevance for diagnosis and therapeutic management

**DOI:** 10.3389/fneur.2023.1201643

**Published:** 2023-08-11

**Authors:** Antonio Lotti, Alessandro Barilaro, Alice Mariottini, Lorenzo Vannozzi, Marco Piergentili, Enrico Fainardi, Luca Massacesi

**Affiliations:** ^1^Department of Neurosciences, Drug and Child Health, University of Florence, Florence, Italy; ^2^Department of Neurology 2, Careggi University Hospital, Florence, Italy; ^3^Department of Ophthalmology, Careggi University Hospital, Florence, Italy; ^4^Neuroradiology Unit, Careggi University Hospital, Florence, Italy

**Keywords:** Susac syndrome, vessel wall imaging, leptomeningeal enhancement, VWI, CE-FLAIR, neuroimaging, vasculitis

## Abstract

**Background:**

Susac syndrome (SS) is a rare immune-mediated vasculitis affecting retina, inner ear and brain. Assessment of central nervous system (CNS) involvement is currently based on standard brain magnetic resonance imaging (MRI) sequences. Accuracy of three dimensional (3D)-vessel wall imaging (VWI) was compared to standard sequences and contrast-enhanced-3D T2-fluid attenuated inversion recovery (CE-FLAIR) to assess CNS disease activity in two cases of definite SS.

**Methods:**

Brain MRI scan and retinal fluorescein angiogram (RFA) were performed at disease onset and at 1, 3, and 6 months after induction therapy start. CE-FLAIR and VWI based on 3D black-blood proton density weighted (PDW) with and without gadolinium were added to standard sequences on a 3 Tesla MRI scanner.

**Results:**

Contrast enhanced-VWI (CE-VWI) detected an abnormal diffuse leptomeningeal enhancement (LME) in both cases at onset and during follow-up. Pathological enhancement on CE-VWI persisted at 6-month brain MRI, despite absence of new lesions and disappearance of LME on CE-FLAIR. Follow-up RFA revealed new arterial wall hyperfluorescence in both cases.

**Conclusions:**

VWI may represent a useful tool for diagnosing and monitoring CNS disease activity in SS patients, as confirmed by concordance with RFA, leading treatment's choice and timing. Moreover, CE-VWI seemed at least as sensitive as CE-FLAIR in detecting LME, possibly being superior to the latter in posterior fossa. LME remission might be not accurate in predicting suppression of CNS inflammation in SS.

## Introduction

Susac syndrome (SS) is a rare immunological disorder affecting retina, inner ear and brain, through an immune-mediated small vessels-occlusion (1). Recent pathological findings confirmed this hypothesis, showing inflammation of small leptomeningeal and parenchymal arterioles, capillaries, and venules (2). Classically, SS presents with the clinical triad of vision disturbances, hearing loss, and encephalopathy (1). The neuroimaging triad, first described in 2003, consists of white matter lesions, deep gray matter lesions, and leptomeningeal enhancement (LME) (3). LME, when investigated with three dimensional (3D) contrast-enhanced (CE) T2-fluid attenuated inversion recovery (CE-FLAIR), is an early finding and always detectable, disseminated at both supratentorial and infratentorial areas (4). Moreover, LME seems an MRI marker useful for monitoring disease activity (5). Linear enhancement in CE-vessel wall imaging (VWI), possibly related to inflammation of small intracranial vessels, was recently suggested as a potential diagnostic biomarker, as described in two recent case reports (6, 7). Assessment of central nervous system (CNS) involvement is currently based on standard brain MRI sequences: the presence of new lesions or persistent enhancement of previous lesions and residual LME are, to date, the only know brain MRI markers of disease activity (8). We herein describe the use of 3D vessel wall imaging with and without gadolinium (VWI) in two cases of definite SS, comparing it with standard sequences and CE-FLAIR to assess CNS disease activity and leading treatment's choice and timing.

## Case 1

**Figure 1:**
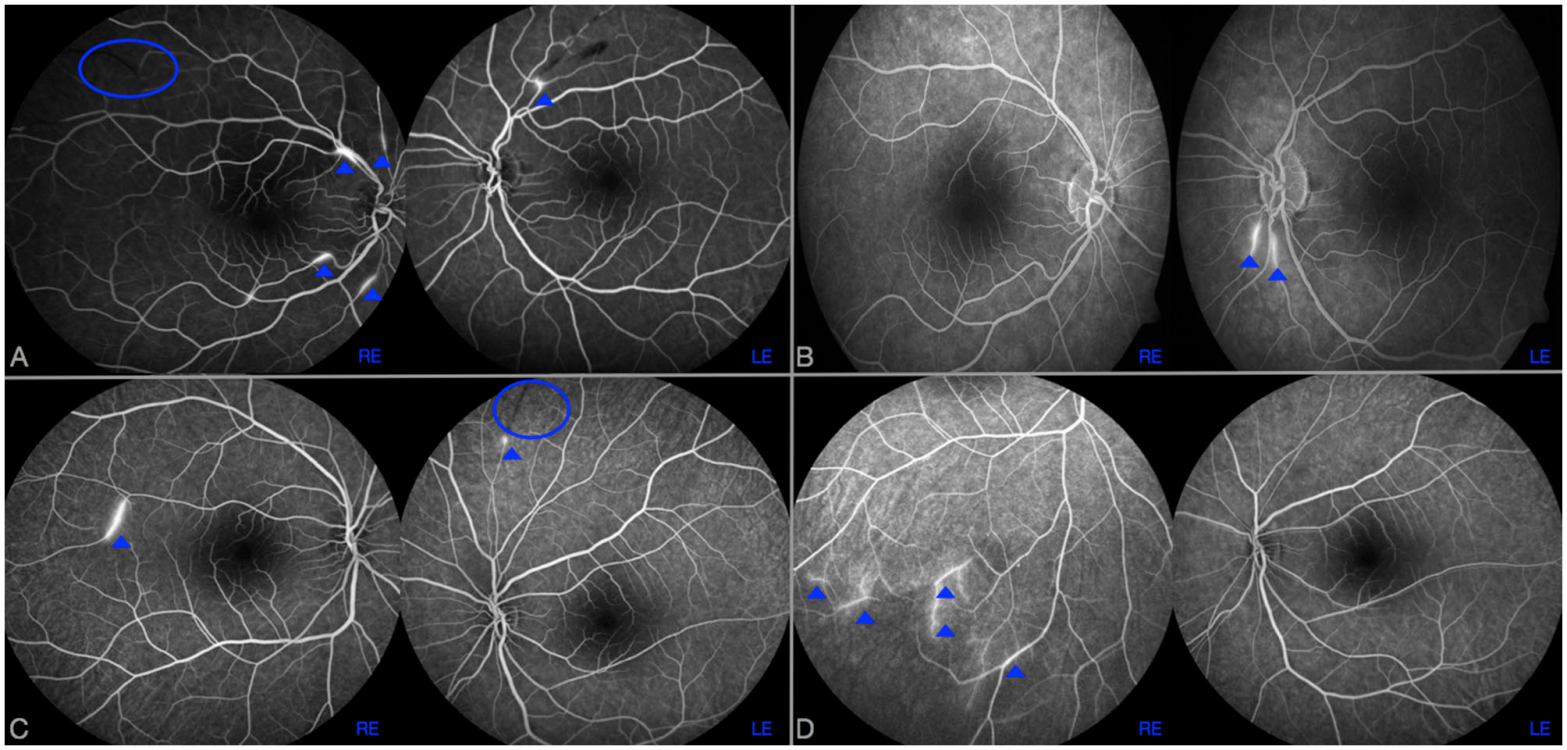
RFA at disease onset **(A, C)** and after 6 months from the start of induction therapy **(B, D)** in case 1 **(A, B)** and 2 **(C, D)**. BRAOs are highlighted by a blue ovoid and AWH by dot arrows. In case 1, despite a complete disappearance of AWH in right eye (RE) after 6 months of cyclophosphamide therapy **(B)**, two new AWH were observed in left eye (LE) compared to the examination performed at disease onset **(A)**. Similarly, in case 2, new AWH in peripherals inferior temporal branches in RE were observed 6 months after rituximab commencement **(D)**; no AWH were observed in LE.

**Figure 2:**
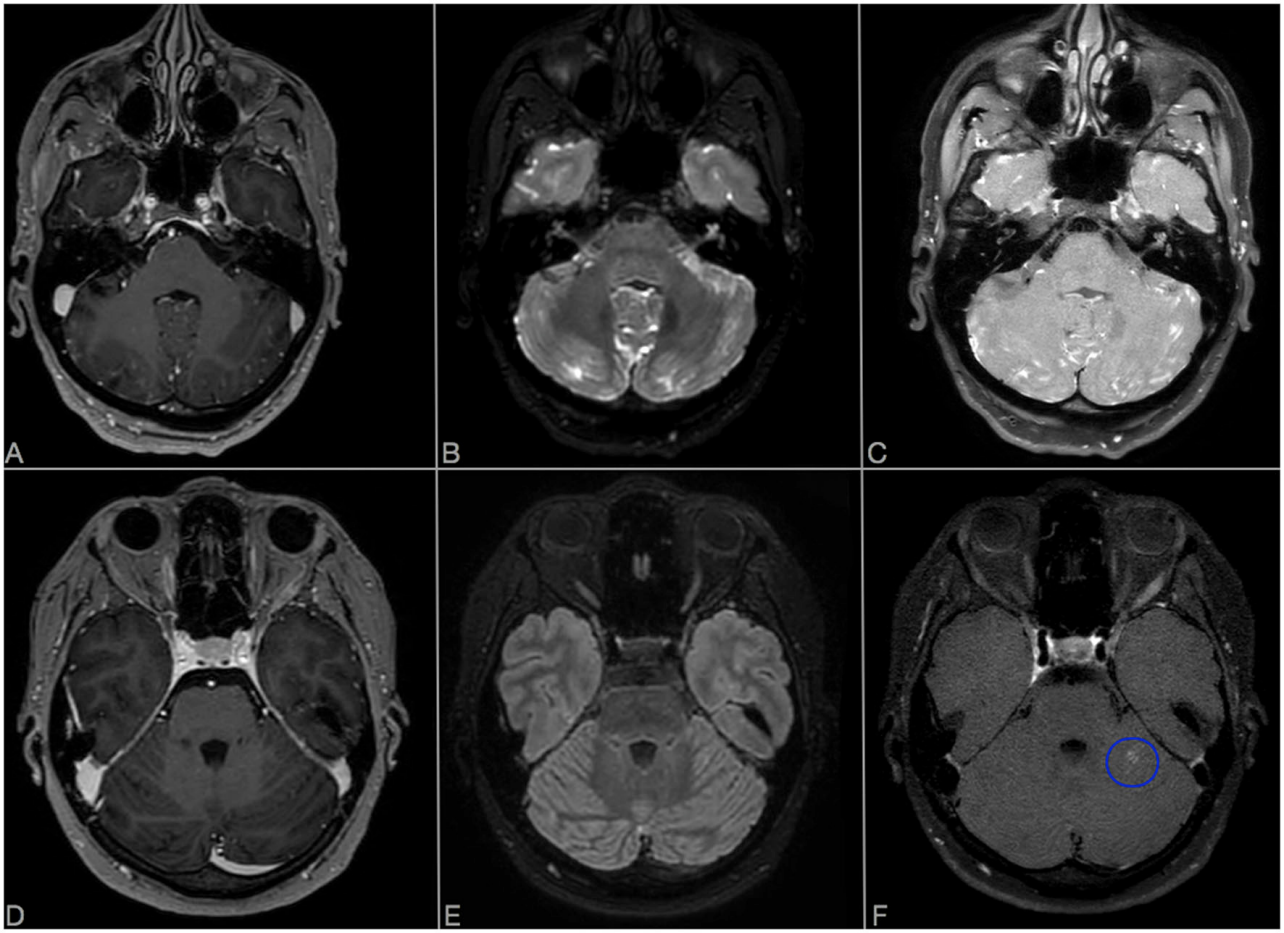
Comparison of axial CE-T1 **(A, D)**, CE-FLAIR **(B, E)**, and CE-VWI **(C, F)** sensibility in detecting LME in posterior fossa. **(A–C)** Show LME evaluation at onset in Case 1: LME is more evident in CE-VWI compared to CE-T1 and also to CE-FLAIR, located along the folia of the cerebellar hemispheres and vermis. **(D–F)** Show evaluation of LME regression in Case 2, 3 months after rituximab: despite CE-T1 and CE-FLAIR did not detect any residual LME, CE-VWI revealed a residual patch enhancement of the folia in left cerebellar hemisphere [blue ovoid, **(F)**].

**Figure 3:**
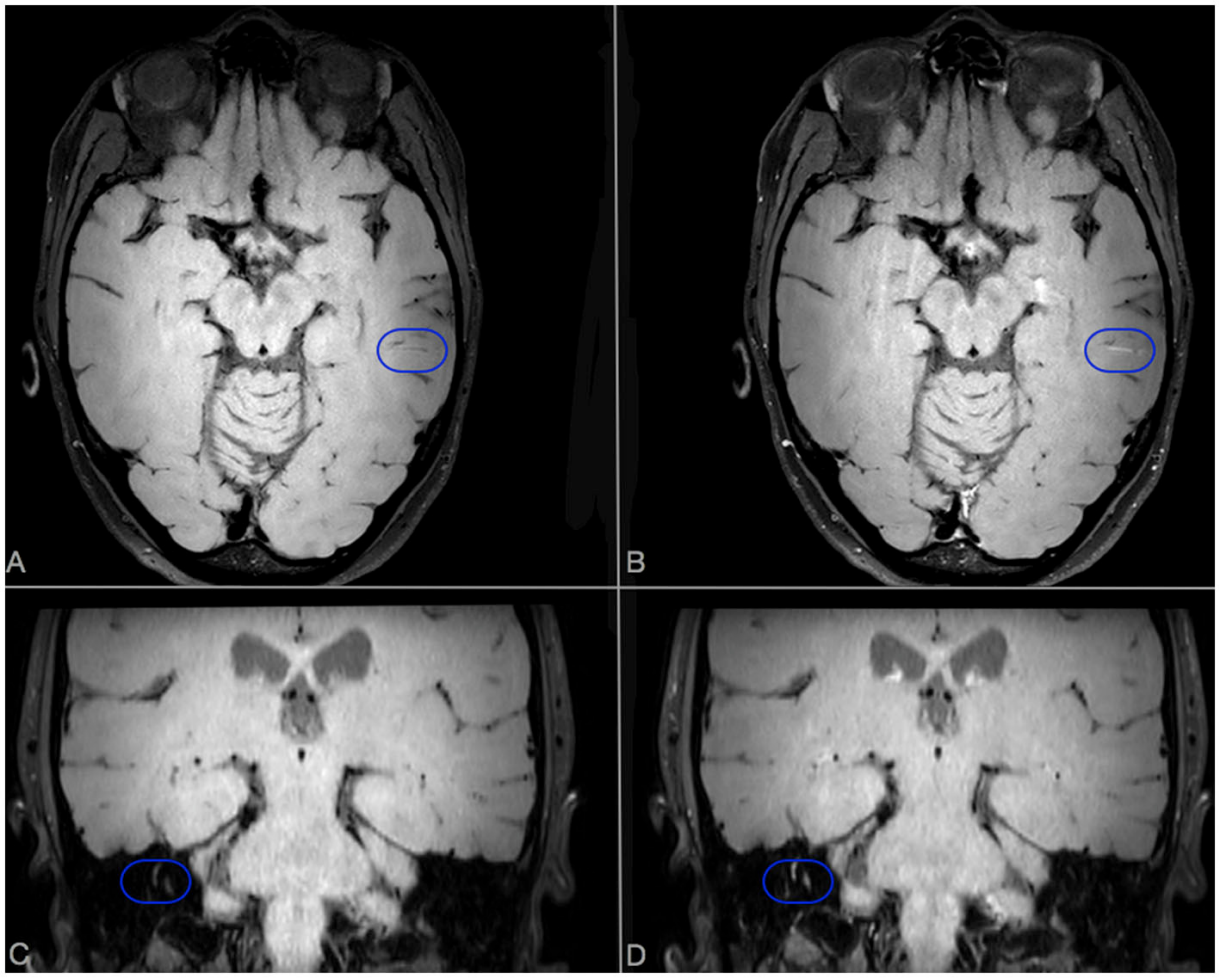
Brain MRI scan performed after 6 months of cyclophosphamide therapy in case 1. Axial VWI sequences without **(A)** and with **(B)** Gadolinium. A linear enhancement, compatible with a persistent inflamed vessel, is evident in CE-VWI [blue ovoid **(B)**]. In coronal scans without **(C)** and with **(D)** Gadolinium, an abnormal enhancement of semicircular canals is detected in CE-VWI [blue ovoid **(D)**].

A 40-year-old woman presented to the emergency department because of sudden onset of visual field loss in her right eye and headache. Retinal fluorescein angiogram (RFA) showed a recent superior temporal branch retinal artery occlusion (BRAO) in her right eye and multiple arterial wall hyperfluorescence (AWH) in both eyes (Figure 1A). She underwent brain MRI that revealed multiple T2 hyperintense foci in the periventricular white matter and corpus callosum with restricted diffusion, most of which gadolinium-enhanced, suggestive for SS. Diffuse LME was detected in CE-T1, CE-FLAIR, and CE-VWI but it was more evident in the two latter, being the LME the posterior fossa more evident in the CE-VWI compared to the CE-FLAIR (Figures 2A–C). Cerebrospinal fluid (CSF) analysis revealed increased protein levels (1.6 g/L), absence of leucocytes, a normal glucose ratio, absence of oligoclonal bands and normal IgG index (<0.7). CSF PCR for neurotrophic viruses (HSV, CMV, VZV, EBV, HHV6, HHV7, HHV8, Enterovirus, Adenovirus, and Toscana Virus), as well as fungal and bacterial cultures were all negative. Her past medical history was unremarkable, excepted for an episode of vestibulocochlear neuritis inducing sensorineural hearing loss in the left ear when she was 12 years old. An audiometry investigation confirmed previous sensorineural hearing loss in the left ear. The patient was treated with a 5-day course of intravenous methylprednisolone 1,000 mg daily, followed by oral prednisone 1 mg/kg and intravenous immunoglobulin (IVIg) 2 g/kg, with clinical improvement. A few days later, she complained of hearing loss in her right ear with evidence of a severe sensorineural hearing loss at audiometry. The clinical triad was then fulfilled, allowing a definite diagnosis of SS (9). A follow-up brain MRI taken 1 month later showed the appearance of at least 15 new T2-hyperintense lesions in the semioval centers. She therefore started an induction immunosuppressive treatment with cyclophosphamide 0.7 g/m^2^ body surface area/month. A third brain MRI, performed 3 months after the first administration of cyclophosphamide, showed evidence of new infratentorial and deep gray matter lesions, with fulfillment of the neuroimaging triad (3). Despite the ongoing treatment, the patient complained of persistent cognitive impairment and worsening of hearing in the right ear. The brain MRI repeated at 6 months after cyclophosphamide start did not show any new lesions, but rather a reduction of pre-existing ones. LME was no more detectable on CE-FLAIR, whereas CE-VWI revealed some thin linear enhancement attributable to inflamed vessel, as well as an abnormal enhancement of the right inner ear structures (Figure 3). A control RFA performed a few days later showed new AWH, confirming that the inflammation was not suppressed yet (Figure 1B). Considering the persistence of small vessels inflammation on CE-VWI and RFA, that was interpreted as a partial response to therapy, we decided to prolong the induction treatment with cyclophosphamide for further 6 months, rather than switching to maintenance therapy as usually reported in clinical practice, with subsequent stabilization of the clinical picture.

## Case 2

**Figure 4:**
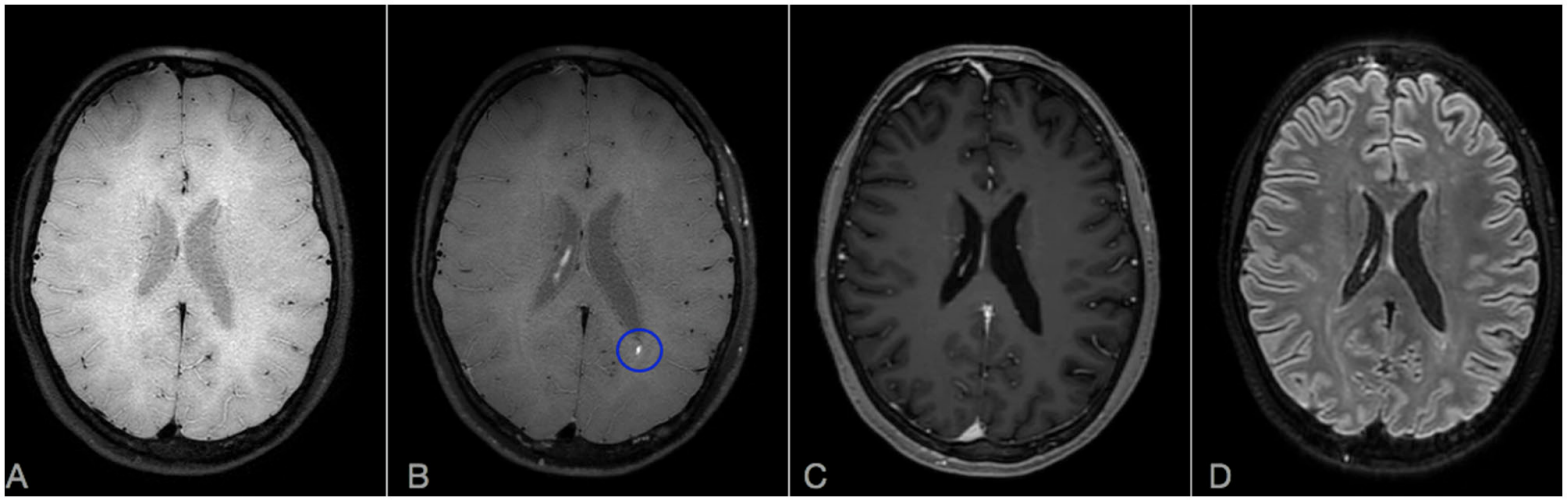
Brain MRI scan after 6 months from rituximab start in Case 2: comparison of CE-T1, CE-FLAIR, and CE-VWI sensibility in detecting residual disease activity. Axial VWI without **(A)** and with Gadolinium **(B)**. **(C)** Axial CE-T1. **(D)** CE-FLAIR. A punctiform persistent enhancement is observable on CE-VWI [blue ovoid, **(B)**] but not in CE-T1 **(C)** or CE-FLAIR **(D)**. Note also that LME is completely absent on CE-FLAIR **(D)**.

A 26-year-old woman presented to the emergency department due to persistent and unusual headache and dizziness, with evidence at brain MRI of multiple T2-hyperintense lesions located in the periventricular white matter, midbrain and central forebrain. Despite the absence of visual symptoms, RFA showed multiple BRAO and AWH in both eyes (Figure 1C). A brain MRI scan with gadolinium was then repeated after 2 weeks, that showed multiple lesions with associated restricted diffusion, most of which gadolinium-enhanced, suggestive for SS. A diffuse LME in both supratentorial and infratentorial areas was also observed on CE-FLAIR and CE-VWI. To complete diagnostic assessment, she performed an audiometry that revealed a mild sensorineural hearing loss in the right ear, thus fulfilling the clinical and instrumental triad of SS. CSF analysis revealed a severe increase in protein levels (2.1 g/L), mild pleocytosis (8/microL), a normal glucose ratio, absence of oligoclonal bands and normal IgG index (<0.7). CSF PCR for main neurotrophic viruses, as well as cultures for fungal and bacterial pathogens were all negative. Her past medical history was unremarkable. Similarly to patient 1, she was firstly treated with a 5-day course of intravenous methylprednisolone 1,000 mg daily, followed by oral prednisone 1 mg/kg and IVIg 2 g/kg with clinical benefit. She was then discharged, planning an induction therapy with rituximab 1,000 mg intravenously 2 weeks apart (10). In the meanwhile, a new MRI brain scan, performed 1 month after onset, did not show any new lesions, but a persistent LME on CE-FLAIR and perivascular enhancement on the frontal and parietal convexity bilaterally on CE-VWI (not shown). One month after the second infusion of rituximab, a slow tapering of prednisone was started, reducing the dose by 10% every 2 weeks. Unfortunately, the patient experienced a sudden visual field loss in her left eye that was associated with a new BRAO at RFA. The following MRI, performed 3 months after start of induction therapy, showed multiple lesions with restricted diffusion in the semioval center despite a regression of LME on CE-FLAIR. Due to the persistence of disease activity, prednisone dosage was increased up to 1 mg/kg and a new course of IVIg was administered, to allow a new attempt of slow steroid tapering until the subsequent infusion of rituximab. The brain MRI control performed at month 6 after rituximab start showed no recent lesions and a complete disappearance of LME in CE-FLAIR, whereas CE-VWI demonstrated some residual punctiform enhancement (Figure 4). The control RFA showed a few new AWH in the absence of recent BRAO (Figure 1D). Such findings were considered as suggestive of persistent disease activity, like in case 1, therefore another infusion of rituximab was planned before switching to maintenance therapy.

## Discussion

Immunosuppression is the cornerstone of SS treatment, and its intensity and duration depend mainly on the severity of CNS involvement (11). Currently, the following are considered as markers of suboptimal therapeutic response: the presence of new or persistently enhancing lesions on brain MRI scan, and new BRAO and/or AWH on RFA, being the latter the most sensitive exam to evaluate disease activity in SS (8). We explored the value of VWI, compared to CE-FLAIR, standard MRI sequences and RFA, to assess disease activity in two cases of SS with prominent brain involvement. VWI based on submillimetric 3D isotropic black-blood proton density weighted (PDW) motion sensitive driven equilibrium with and without gadolinium, were added to standard MRI protocol including also 3D T1 and CE-T1 submillimetric turbo field echo and CE-FLAIR submillimetric volume isotropic turbo spin echo acquisition. MRI scans were all performed on the same 3 T scanner (Ingenia, Philips Healtcare). After gadolinium injection, the sequences were routinely acquired in the following order, with few exceptions: CE-T1, CE-FLAIR, and CE-VWI. Acquisition time was about 3 min for CE-T1, 3 min and 25 s for CE-FLAIR and 5 min and 40 s for CE-VWI. A brain MRI scan and RFA were performed at disease onset and at 1, 3 and 6 months after induction therapy start. In our two cases we demonstrated that the stability of lesion load detected at standard MRI examination is not enough accurate to establish absence of disease activity in the CNS, and that the use of VWI may add valuable information for SS monitoring. Despite absence of new lesions, or even a reduction in lesion number (as in case 1) at standard examination, a pathological enhancement was detected with CE-VWI in both cases, and this was consistent with evidence of disease activity at RFA, suggesting that VWI could be more accurate than standard MRI monitoring in detecting CNS disease activity in SS. In addition CE-VWI, might better reflect the underlying pathogenesis of the syndrome (2). LME assessment on CE-FLAIR is considered another tool useful to diagnosis and monitoring of disease activity in SS, but its regression did not seem sufficient to predict a complete suppression of CNS involvement, as suggested in case 2. Moreover, in our cases, CE-VWI seems to be at least as sensitive as CE-FLAIR in detecting LME compared to CE-T1, and may even be superior to CE-FLAIR in posterior fossa (Figures 2A–F), where LME is more specific for SS (5). These findings appears independent from the order and time of acquisition of VWI sequences after gadolinium injection. Furthermore, in the present case and in another recently published case report of SS (7), an abnormal enhancement of the inner ear was observed using these sequences, revealing an additional finding that may be helpful for SS diagnosis and monitoring disease activity at this level. It is not clear why the enhancement of inner ear was detected in CE-VWI only, but similar findings were observed in other conditions (12) and although not specific it could be useful to assess the involvement of inner ear during the follow-up and to consequently adapt therapy.

## Conclusions

In conclusion, our experience supports the use of VWI as a potential additional tool that may help in the diagnosis of SS with prominent brain involvement. Furthermore, it may be useful in monitoring disease activity and treatment response, supporting therapeutic decisions. CE-VWI seemed at least as sensitive as CE-FLAIR in detecting LME, being also superior to the latter in posterior fossa. The complete remission of LME might be not sufficient to predict the suppression of disease activity in the CNS. To our knowledge, this is the first report describing the use of CE-VWI to monitor residual CNS disease activity in SS, and comparing it to the gold-standard RFA. Despite these encouraging observations, we currently do not suggest using CE-VWI alone, but rather adding it to CE-T1 and CE-FLAIR to better characterize CNS disease activity in SS, integrating different information deriving from multimodal imaging. Further prospective investigation on large cohorts of patients are needed to confirm our findings.

## Data availability statement

The original contributions presented in the study are included in the article, further inquiries can be directed to the corresponding author/s.

## Ethics statement

Written informed consent was obtained from the participant/patient(s) for the publication of this case report.

## Author contributions

AL drafted the manuscript and analyze MRI data. EF analyzed and revised the MRI data. LV and MP analyzed RFA data. AB, AM, and LM revised the manuscript for intellectual content. All authors contributed to the article and approved the submitted version.
